# Visual Impairment among Primary School Children in Gondar Town, Northwest Ethiopia

**DOI:** 10.1155/2020/6934013

**Published:** 2020-08-22

**Authors:** Ayanaw Tsega Ferede, Destaye Shiferaw Alemu, Alemayehu Desalegn Gudeta, Haile Woretaw Alemu, Mulusew Asferaw Melese

**Affiliations:** ^1^University of Gondar, College of Medicine and Health Sciences, Department of Optometry, Northwest Ethiopia, Amhara National Regional State, Gondar, Ethiopia; ^2^University of Gondar, College of Medicine and Health Sciences, Department of Ophthalmology, Northwest Ethiopia, Amhara National Regional State, Gondar, Ethiopia

## Abstract

**Background:**

An impairment of the visual system at or shortly after birth adversely affects educational performance of children which typically occurs through vision. Limited evidence on the magnitude and causes of visual impairment is one of the reasons for the low priority given to eye care in low-income countries.

**Objectives:**

To estimate the prevalence and determine the causes of visual impairment in primary school children in Gondar town, Northwest Ethiopia.

**Materials and Methods:**

A descriptive cross-sectional study was conducted among 1289 children aged 5–15 years who were randomly selected in 9 primary schools (government and private) from May to June 2016. Visual acuity was measured at 6 m using Snellen's chart, and children with an acuity of less than 6/18 in the better eye underwent refraction and a detailed eye examination. A cause of their impairment was determined. Data were recorded using pretested tools. . Statistical Package for Social Sciences version 16 was used to enter and analyze the data using 95% confidence intervals.

**Results:**

The prevalence of visual impairment was 1.8%. Refractive errors (nearly 70%) followed by strabismus and cataract, each contributing 4.3%, were the most frequent causes of visual impairment in the study population. Majority (87%) of the children had moderate degree of vision impairment, and 10–15-year age groups are the more affected ones. Children of age fifteen and above showed statistically significant association with visual impairment (*p*=0.005).

**Conclusion:**

The magnitude of visual impairment in primary school children in the study area is significant. School screening programme is recommended to minimize the burden of visual impairment in the study area.

## 1. Background

Visual impairment is defined as a functional limitation of the eye(s) or the visual system and can manifest as reduced visual acuity or contrast sensitivity, visual field loss, photophobia, diplopia, visual distortion, visual perceptual difficulties, or any combination of the above. Vision impairment ranges in severity from mild visual loss to total absence of light perception [1, 2].

Recently, world report on vision showed that at least 2.2 billion people have a vision impairment or blindness globally and at least 1 billion of whom have a condition which could have been either prevented or has yet to be addressed. According to the World Health Organization (WHO) 2010 reports, approximately 19 million children below 15 years of age are estimated to be visually impaired worldwide [2, 3]. The WHO estimated that 80% of blindness is preventable, but still there are more than 419,000 blind children in Africa and many more with visual impairments, most of which could have been prevented [4]. Research studies in Africa showed that prevalence of visual impairment in children varies from 1.2% to 29.4% [5–8]. In Ethiopia, the proportion of school children with vision impairment ranges from 1.75% to 8% [9–12], whereas the proportion with uncorrected refractive error varies from 4% to 10.2% [13, 14]. Uncorrected refractive error accounts for 77.3% of visual impairment in children [9].

An impairment of the visual system at or shortly after birth adversely affects educational performance which typically occurs through vision. Children who got visual impairment early will have longer life of suffering with the condition [4]. It can also affect areas of gross and fine motor skills, visual perception, and reading at the chalkboard or overhead projection, with inability to discriminate colors which have a significant impact on child's quality of life. More obvious signs of visual impairment include squinting, sitting very close to the chalkboard or screens, messy work, or difficulty of seeing things clearly [15–17].

According to the 2019 world report on vision among children, the causes of vision impairment vary considerably across countries. For example, in low-income countries, congenital cataract is a leading cause, whereas in high-income countries, it is more likely to be retinopathy of prematurity [2]. In Africa, a report on child eye health indicated that the major causes of child blindness and visual impairment are diverse and context-specific. The most common causes are corneal scaring from measles, vitamin A deficiency, the use of harmful traditional eye remedies, and swelling of child's eyelids due to infection [4, 18].

The literature reviewed in school- and community-based studies across different countries in African and Asian regions also showed factors associated with visual impairment. For example, vitamin A deficiency [18, 19], refractive errors [20], corneal abnormalities [7, 21, 22], lens and optic nerve disorders [7, 21–23], ocular trauma [24], and congenital cataract [25] were reported as causes of visual impairment.

There are different strategies or interventions at different levels of visual conditions to reduce the impact of visual impairment on the child. Some are among the most feasible and cost-effective ones to implement. For example, the uncorrected refractive error can be corrected with glasses, while surgery for cataract and eye patching for amblyopia are best interventions to restore vision. Vision rehabilitation services are also effective in improving function of people with irreversible vision impairment [2]. Academically and socially, many of them appear to be quite successful [16].

Despite all these reasons and hopes for restoring vision, there are limited data about magnitude visual impairment in school children in Ethiopia. Particularly in the study area, there is only one previously published study focused on the prevalence of refractive error among all age groups of students and it does not show the magnitude of visual impairment in school children. In addition, the results will be important to prioritize resource allocations, to see regional variations, as compared with the reported prevalence of visual impairment in studies performed in Ethiopia in different zones at community level. Therefore, this study was aimed to generate evidence by determining the burden of visual impairment in school children in Gondar town.

## 2. Materials and Methods

### 2.1. Study Design, Setting, and Population

A cross-sectional study design was employed to determine the prevalence and causes of visual impairment in primary school children in Gondar town from May to June 2016. One thousand two hundred eighty nine (1289) children aged 5–15 were randomly selected from 9 primary schools (6 government and 3 private) using multistage stratified random sampling technique. Data on the total number of private and government primary schools (*n* = 58) and of students registered (61,560) in the new academic year were obtained from the Gondar town district educational office. First, nine schools out of 58 were determined by chance. Then, the total sample size (*n* = 1289), which was determined using the formula for a single population proportion, was proportionally allocated to the selected schools. Finally, using a systematic sampling technique (sampling fraction, *K* = *N*/*n*), children were included from each school until proportionally allocated sample is achieved. The first child in the class was randomly selected using the lottery method, and the next child was included by keeping the sampling fraction. Children whose consent/assent were obtained and attended their class on the date of data collection were included. Children in night schools and attended school for different reasons were excluded.

### 2.2. Data Collection Tool and Instruments

Data were collected by qualified optometrists using standardized check list and physical examinations. Optometrists measured visual acuity including pinhole vision by Snellen's charts and performed the refraction using trail sets and retinoscope. . Children with a visual acuity of less than 6/18 in one or both eyes underwent ocular examination. Refraction was undertaken in a class room with appropriate illumination. Torchlight, direct ophthalmoscope, and handheld slit lamp bio-microscope were used to examine anterior and posterior ocular structures by an ophthalmologist.

#### 2.2.1. Operational Definitions

  Visual impairment was considered as a presenting distance visual acuity of less than 6/18 in the better eye measured by Snellen's chart at 6 meters
   Refractive error was defined if a child has myopia of <0.50 D, hyperopia of >+1.00 D, and/or astigmatism (>1.00 DC) in subjective refraction

#### 2.2.2. Data Management and Analysis

Coded data were entered into SPSS version 16 for analysis. Descriptive statistics was used to analyze the result. Results were presented using tables and texts.

## 3. Results

### 3.1. Sociodemographic Characteristics of Study Population

A total of 1289 primary school children (55.2% are females) from nine primary schools were included in the study, i.e., response rate of 100%. The mean age was 10.83 (SD ± 2.44, range 5–15) years. The majority of the children were aged 10 to 15 years. The majority (85.7%) of children are from government schools.

### 3.2. Prevalence and Degree of Visual Impairment

The prevalence of bilateral visual impairment in the study population was 1.8%, while the prevalence of uniocular visual impairment was found to be 3.7% using vision <6/18 in either eye definition. Twelve (52%) male children were bilaterally visually impaired though not statistically significant (*p* > 0.05). The majority (98.2%) of children have normal-to-mild vision impairment (6/6–6/18), followed by moderate visual impairment in 1.6% (<6/18–6/60). There was no severe visual impairment in the study population. Among students with moderate visual impairment, females accounted for about 55%. There were 47 eyes (3.65%) that are visually impaired among school children (see Table 1).

### 3.3. Causes and Proportion of Visual Impairment

The most frequent cause of uniocular and bilateral visual impairment in the study population is refractive error (Table 2). More than two-thirds of proportion of both uniocular and bilateral visual impairments were due to refractive errors. There were three blind children enrolled with normal sight but use Braille and other techniques to learn and cope with friends.

Children's age was the sociodemographic variable that showed statistically significant association with visual impairment (*p*=.0.044). Children of age fifteen and above showed statistically significant association with visual impairment (*p*=0.005). Without taking other variables into account, children of age 15 and above were more than 6 times (COR: 6.18, 95% CI, 1.75–21.9) to be visually impaired than those children in the age between 5 to 10 years. There was no statistically significant difference in visual impairment between males and females (*p*=0.47).

## 4. Discussion

Globally, visual impairment and blindness are among the major public health issues especially in developing countries such as Ethiopia. Knowing the magnitude and causes of the disease will help to plan action to reduce the impact at individual, community, and national levels through evidence-based implementation of treatment and prevention strategies for avoidable causes.

As a result, this study was conducted in Gondar town among children and found that the prevalence of visual impairment was 1.8%. This result is in agreement with the study performed in the southern part of Ethiopia, Goro district (1.75%) [10]. The probable reason could be the similar age group of population. On the other hand, this finding is much lower to studies in other parts of Ethiopia. For example, in Amhara regional state, Bahir dar city, and Sekela district, visual impairment was reported to be 8.7% and 8%, respectively [12, 26], and in the capital city, Addis Ababa, two studies showed 5.8% and 7.24% [9, 11]. In addition, the two studies in Amhara region reported higher prevalence of visual impairment and this is true that there is high chance of detecting children with the condition at community level.

Using the definition of the better eye's vision or either eye's vision less than 6/18, this result is found to be higher than that in Addis Ababa. Arada subcity reported results of 0.53% and 1.1% [9] and one population-based study in the southwest, Sekuro district, showed results of 0.062% [25]. The difference might be the sample size selection criteria; that is, the latter study showed that children included were already either blind or with severe visually impaired or just undergone for examination to identify the causes, but in the current study, there was no predetermined selection of children, i.e., chance was given to all. The reason could be the characteristics of population differences for eye care services.

However, the prevalence in the study area was lower than other study reports of developed countries such as Egypt (29.4%) [27], Northern Ireland (3.6%) [21], Nepal (9.1%) [22], Brazil (2.67% and 4.82%) [28, 29], two regions of China (6.37% and 7.7%) [30, 31], and Australia (6.4%) [32]. This is probably due to variation in the measurement of visual impairment, where in this study, children presenting with a visual acuity of <6/18 in the better eye were considered as visually impaired, whereas majority of the above used children presenting with a visual acuity of less than or equal to 6/12 and specifically, vision ≤6/9 was used in Egypt. In addition, the discrepancy could also be attributed to the difference in age of children included in the studies, in which some studies included children up to age of 18 years since increasing age is an important predictor of visual impairment. Some of the studies are population-based house-to-house studies which may include preschoolers and children dropped out school for various undetected ocular conditions.

Majority (87%) of the school children in the current study were with moderate visual impairment, and age groups of 10–15 years were the more affected ones. This implies that vision recovery could be possible if refractive errors are detected and treated early. In Egypt, the proportion of moderate visual impairment was 2%. However, in the current study, there were no children with severe visual impairment and only 3 (0.2%) children were found to be bilaterally blind. In contrast, a study performed in schools for the blind in Eastern Africa reported that about 65.2% them are with severe visual impairment or blind [7]. It can be understood here that children in schools for the blind are already identified as having visual impairment and enrolled in separate schools, while children found visually impaired in this study and other studies are expected to have healthy and normal vision.

Reviewed studies conducted at different time points and towns in Ethiopia showed the prevalence of refractive errors as 4%, 9.3%, and 10.2% [13, 14, 33]. The ocular condition is still common in some parts of the world. For instance, prevalence data were reported as 4.4% in Nigeria, 19.2% in Vietnam, and 20.69% in China [5, 30, 34]. It can be said that the magnitude is significant in countries with reported data, and the possible explanation could be that environmental factors will contribute to the development of refractive errors especially at school age and spending more time indoors and excessive near work will lead to myopia onset [35]. Specifically, the probable scientific reason for high prevalence in China could be the genetic influences and geographical variations.

Uncorrected refractive errors are found to be the main cause for 70% of visual impairment in children in this study. This is supported by reports in Addis Ababa, Ethiopia (70.3% and 77.3%), Sudan (36%), Vietnam (92.7%), Nepal (93.3%), China (86%), and Australia (69%) [8, 9, 11, 22, 31, 32, 34]. In addition, the 2019 WHO report indicated that refractive error still remains the number one cause of visual impairment in the world. This may be due to lack of awareness about the symptoms and use of eye glasses for correction. Besides, uncorrected refractive error is painless visual condition and children with it may live without complain to parents and may not seek eye care as compared with children with infectious eye disease. Despite variations discussed in the above-reported prevalence of visual impairment and refractive errors, majority of the findings indicate the need to focus on reducing uncorrected refractive errors and then visual impairment by taking child eye health as public health agenda.

In addition to refractive errors, strabismus (unilateral visual impairment) and cataract were second and third causes of visual impairment. Cataract is reported as the main cause in African countries [8, 36] and is reported in small percentage in developed countries. However, in other studies, amblyopia [9, 29, 31, 34] becomes the second leading cause of visual impairment in school children followed by retinal disorders. This variation could be attributed to variations in distribution of ocular problems across different countries based on socioeconomic development. Retinal disorders such as optic nerve lesions and retinopathy are commonly found in developed countries, but corneal opacities from infections disease and xerophthalmia are less common in developed countries.

The odds of being visually impaired with age above 15 years was 6 times more likely than the counterparts with a crude odds ratio of (COR) 6.18 (95% CI, 1.75–21.9). A single study in Ethiopia similarly reported that children of age 10 and above are almost 3 to 4 times more likely to be affected by visual impairment [11]. This finding supported and strengthened the previous fact that visual impairment will be more with increasing age as frequent evidence was presented and reported by the WHO and IAPB. However, no statistically significant gender difference was shown with visual impairment in this study.

One of the limitations of this study is that the findings might not show the causal relationship between visual impairment and other independent variables because of the nature of the method used. Recently, vision of 6/12 in either eye is used as the cutoff point for definition of visual impairment and refractive error. But this study reported vision less than 6/18 in the better eye as the cutoff point, and therefore, there might be underreporting of both visual impairment and refractive errors in children. Also, the result may not be generalized to children who do not have access to school due to different reasons, and the result may not show the real picture of the problem at community level.

## 5. Conclusion

Visual impairment in the study area is a significant public health problem in school children. Refractive errors are the major causes of the problem. Hence, priority should be given to start school vision screening in primary school children in the study area.

## Figures and Tables

**Table 1:** Proportion and degree of visual impairment in terms of sociodemographic variables among visually impaired children in Gondar town.

Characteristics		Uniocular visual impairment (*n* = 47, 3.65%)	Bilateral moderate and severe VI (<6/18–3/60) (*n* = 23)	Blind (<3/60)
Sex	Male	17 (36.2%)	9 (39.1%)	3 (13.1%)
	Female	30 (63.8%)	11 (47.8%)	0%

Age group	5–10 years	12 (25.5%)	5 (13.1%)	2 (8.7%)
	10–15 years	35 (74.5%)	15 (73.9%)	1 (4.3%)

Laterality	Right	24 (1.9%)	20 (1.6%)	3 (0.2%)
	Left	23 (1.8%)		

**Table 2:** Cause-specific proportion of visual impairment among visually impaired children in Gondar town.

Ocular condition	Unilateral visual impairment/blindness (*n* = 47)		Bilateral visual impairment/blindness (*n* = 23)	
	*N*	Percentage	*N*	Percentage
Refractive error	33	70.1	18	78.3
Strabismus	1	2.1	0	0
Cataract	3	6.4	1	4.3
Corneal opacity and cataract	2	4.3	1	4.3
Glaucoma	2	4.3	0	0
Retinal disorders	1	2.1	0	0
Corneal trauma	2	4.3	0	0
Unconfirmed	3	6.4	3	13
Total	47	100	23	100

## Data Availability

The data in which this result finding is based are available in soft copy with the corresponding author. Any query related to data can be deciphered by contacting the corresponding author (e-mail: ayanaopto@gmail.com).
